# Sepsis Alerts in the Pre-hospital Setting: An Observational Retrospective Study of Emergency Medical Services’ Response in Portugal (2020–2023)

**DOI:** 10.7759/cureus.82528

**Published:** 2025-04-18

**Authors:** Adelaide Moutinho, Joana Fontes, Lénia Ferreira, José Lopes, Fábio Martins, Solange Mega, Margarida Gil, Filipa Barros, Ana Margarida Correia

**Affiliations:** 1 Internal Medicine: Sepsis Care Process, Instituto Nacional de Emergência Médica, I.P., Porto, PRT; 2 Nursing, Sepsis Care Process, Instituto Nacional de Emergência Médica, I.P., Porto, PRT; 3 Nursing, Sepsis Care Process, Instituto Nacional de Emergência Médica, I.P., Coimbra, PRT; 4 Nursing, Sepsis Care Process, Instituto Nacional de Emergência Médica, I.P., Lisboa, PRT; 5 Nursing, Sepsis Care Process, Instituto Nacional de Emergência Médica, I.P., Faro, PRT; 6 Epidemiology and Public Health, Department of Coordination of the Integrated Medical Emergency System, Instituto Nacional de Emergência Médica, I.P., Lisboa, PRT; 7 Internal Medicine, Department of Coordination of the Integrated Medical Emergency System, Instituto Nacional de Emergência Médica, I.P., Lisboa, PRT

**Keywords:** emergency medical services, pre-alert, pre-hospital, sepsis, sepsis management

## Abstract

Background

Sepsis is a life-threatening condition that demands prompt recognition and intervention to enhance patient outcomes. Early identification and timely treatment, particularly in the prehospital setting, are essential.​

Objective

This study aims to characterize sepsis pre-alerts issued by the Portuguese Emergency Medical Services (EMS) early warning system between May 2020 and December 2023, focusing on adult patients. It provides an overview of the alert system and examines associated clinical data, therapeutic interventions, and hospital referrals.​

Methods

A retrospective analysis was conducted on sepsis pre-alerts from the Portuguese EMS database. Data collected included patient demographics, comorbidities, National Early Warning Score (NEWS), interventions administered, and outcomes.​

Results

A total of 537 sepsis alerts were identified, with a median patient age of 83 years. The majority of patients had significant cardiovascular and neurological comorbidities. The average NEWS was 11.74. Advanced Life Support (ALS) or Integrated Life Support (ILS) teams were required in 76.9% (N=413) of cases. Interventions included intravenous fluid administration in 49.3% (N=265), oxygen therapy in 46.2% (N=248), and vasopressor use in 3.9% (N=14).

Conclusions

Effective prehospital sepsis management is crucial for improving patient outcomes. Challenges such as delayed hospital transfers, often due to regional constraints, highlight the need for enhanced integration between EMS and hospital care. Future efforts should focus on optimizing early sepsis management, fostering collaboration between EMS and hospital teams, and exploring the feasibility of prehospital antibiotic administration.

## Introduction

Sepsis is a life-threatening condition characterized by organ dysfunction due to a dysregulated host response to infection, often leading to organ failure and death [1-3]. It represents a significant global challenge, with an estimated 30 million cases and around 6 million sepsis-related deaths annually, with mortality rates ranging from one in three to one in six of those affected [1,4].

Early identification and appropriate management with antibiotics and fluid resuscitation in the initial hours after the onset of sepsis improve outcomes [1-3,5,6].

Emergency Medical Services (EMS), as in other time-sensitive cases (cardiac arrest, stroke, trauma, and myocardial infarction), provide an excellent opportunity for early sepsis recognition and medical intervention. Early recognition improves outcomes by signaling these patients, initiating fluid resuscitation, providing organ failure support, and sometimes transferring them to referral centers that can provide the indicated support to septic patients [2,5,7].

In Portugal, to the best of our knowledge, there are no published studies on sepsis patients in the pre-hospital setting. Therefore, as there is an early detection system in place, it is crucial to understand the outcomes for these patients.

The study aims to characterize the sepsis pre-alerts generated in Portugal according to national guidelines [8] from May 2020 to December 2023, focusing on adult patients. Secondarily, the study aims to present our EMS alert system, describe the demographics and clinical aspects of the alerted patients, evaluate the therapeutic approaches used by pre-hospital teams, and assess hospital referrals.

This article was previously presented as a poster at the 9th National Emergency Meeting on October 4, 2024, organized by the Portuguese Society of Internal Medicine.

## Materials and methods

Study setting

In Portugal, pre-hospital care is coordinated by the Instituto Nacional de Emergência Médica (INEM) [9], a public organization that, among other duties, is responsible for handling health emergency 112 calls and dispatching the proper EMS teams to medical incidents. These teams are organized as basic life support (BLS) teams, involving specialized INEM technicians, firefighters, and Red Cross teams, integrated life support (ILS) teams, consisting of nurses and specialized INEM technicians, and advanced life support (ALS) teams, formed by physicians and nurses. The BLS and ILS teams operate with ambulances to evaluate and transport patients. INEM also provides four emergency helicopters with ALS teams that can evaluate and evacuate patients from remote areas of Portugal. Antibiotics are not available in the Portuguese pre-hospital setting. Fewer than 10% of ALS teams are equipped with point-of-care devices for serum lactate measurement, a key biomarker in sepsis.

All emergency medical teams follow national guidelines with procedure and treatment protocols for specific symptoms or diagnoses, in real time, under the supervision of coordinating physicians at the National Medical Emergency Coordinating Centre (CODU), who oversee decision-making and ensure the quality and safety of the EMS approach. The CODU´s coordinating physician identifies the most appropriate hospital for referral and contacts the hospital, pre-alerting and communicating the arrival time of critical patients, ensuring continuity of care.

In Portugal, emergency health-related calls to 112 are assessed at the CODU, where an algorithm-based triage determines the priority level and dispatches the appropriate team: the more serious cases, named P1, trigger the dispatch of an ALS or ILS team, while the less severe situations, named P3, lead to the deployment of a BLS team.

Sepsis alerts are generated on-site by our prehospital teams through a digital platform (INEM Tool for Emergency Alert Medical System - iTeams™). Nevertheless, access to this platform is not yet available to all BLS teams. Since its implementation around 2020, the alert system has been gradually modified. At first, it only captured alerts that strictly followed the national guidelines [8]. However, a comparison with epidemiological data highlighted that the initial criteria failed to capture a substantial proportion of sepsis cases. Therefore, since 2024, the Portuguese EMS sepsis alert uses the same combination of factors determined in the guidelines (hypo or hyperthermia (Temperature (T) <35ºC or T >38ºC), low systolic blood pressure (sBP <90 mmHg), reduced peripheral oxygen saturation (SpO_2_ <90%), or mental status alteration), but broadened the spectrum changing the combinations from “and” to “or”, while still maintaining infection suspicion (cough/dyspnoea, abdominal pain, cutaneous inflammatory changes, dysuria/polyakiuria) as the trigger.

According to these inclusion criteria, the clinical record platform produces a visual alarm simultaneously in the EMS team tablet and at the CODU. Upon receiving the alert, the coordinating physician evaluates for exclusion criteria and pre-notifies the hospital.

The emergency departments of Public Portuguese Hospitals can be classified as basic, medical-surgical, or polyvalent. The basic emergency departments have most frequently general physicians working, limited laboratory analyses, most with no access to CT, and a few without access to antibiotics. Medical-surgical emergency services are staffed at least by internal medicine, orthopedics, general surgery, and pediatrics physicians and nurses, and have no restrictions on diagnostic tests; most do not have intensive care units. Polyvalent emergency services have intensive care units and most medical specialties (including neurosurgery, plastic surgery, ophthalmology, and vascular surgery).

Finally, it is also important to note that, since hospitalization in the private sector - whether in general wards or intensive care units - is expensive, transfers from private emergency departments to public hospitals are frequent. These transfers are primarily coordinated by INEM since the public healthcare system provides care free of charge to patients.

Data collection and statistical analysis

We conducted a retrospective and descriptive study of all EMS sepsis alerts generated by the iTeams™ application between May 1, 2020, and December 31, 2023. The data were compiled into a national database, exported, and analyzed with Microsoft Excel™ (Microsoft Corporation, Redmond, WA, US). National guidelines were then applied to standardize the selected alerts [8], taking into account the change in trigger criteria explained above. Individuals under 18 years of age were also excluded from the study. Demographic factors, clinical reports, and National Early Warning Score (NEWS) were analyzed descriptively. Furthermore, we considered the complexity of the team involved in analyzing the therapeutic approaches applied.

## Results

A total of 537 suspected sepsis alerts were analyzed. The characterization of the events is shown in Table 1. The highest number of cases was recorded in 2023, totaling 199 (37.1%). In 76.9% of sepsis alerts (N=413), the CODU triage categorized them as P1, leading to the dispatch of an ALS team in 15.9% (N=85) of cases and an ILS team in 50.7% (N=272). In 56 cases (10.%), despite the severity, only a BLS team was dispatched, as it was the only available team. Regarding symptoms suggesting the source of infection, respiratory symptoms were the most common (50.7%, N=272), followed by impaired consciousness (30.9%, N=166) and urinary symptoms (6.7%, N=36).

**Table 1:** Characterization of sepsis alert events Descriptive data has been represented as N, %. Time data has been described as the median in minutes. 1 – Includes data from May to December; 2 – Time zero being the emergency call; 3 – Given N=418, radio information of arrival to the patient; 4 – Given N = 343, radio information of arrival to the hospital. ALS: Advanced Life Support; ILS: Integrated Life Support; BLS: Basic Life Support

Variable	
Sepsis alerts (N)	537
Year of the event (N, %)	
2020	55 (10.2)^1^
2021	129 (24.0)
2022	154 (28.7)
2023	199 (37.1)
Triage result (N, %)	
P1	413 (76.9)
P3	124 (23.1)
Type of team involved (N, %)	
ALS	85 (15.9)
ILS	272 (50.7)
BLS	180 (33.5)
Time (median, minutes)	
Symptoms onset – emergency call	90
Arrival of the EMS team at the patient^2^	26
Approaching the patient^2^	28
Arrival at the hospital^2^	85
Total^2^	109
Time (N, %)	
Arrival at the patient within the 15-minute mark^2,3^	48 (6.9)
Arrival at the hospital within the 60-minute mark^2,4^	41 (7.6)

Regarding the origins of the patients, while the majority resided at home (28.3%, N=152), 25.7% (N=138) lived in elderly care facilities, and 3.0% (N=16) were directly approached from healthcare establishments, including private hospitals, clinics and secondary transfers of septic patients from non-specialized to polyvalent emergency departments.

The demographic characteristics and patient background are presented in Table 2. There were no significant gender discrepancies, as 50.1% (N=269) of the subjects were male. The median age was 83 years, with 86.0% (N=462) of the individuals being over 65 years old.

**Table 2:** Description of the demographic features and patients’ background Descriptive data have been represented as N, %. Age data have been represented as median in years. 1 – Indicating that the appropriate field for specifying this information was not completed; 2 – Some of the diseases with incidence higher than the 15% found in our population.

Variable	
Gender	
Male	269 (50.1)
Female	268 (49.9)
Age (median, years)	83
≥65 years old (N, %)	462 (86.0)
Patient’s location (N, %)	
Patient’s residence	152 (28.3)
Elderly institution	138 (25.7)
Health facility	16 (3.0)
Public places	11 (2.0)
No record^1^	220 (40.3)
Medical history^2^ (N, %)	
Cardiovascular diseases	319 (59.4)
Arterial hypertension	229 (43.5)
Dysrhythmia	94 (17.8)
Heart failure	90 (17.1)
Neurological diseases	214 (39.9)
Dementia	160 (30.4)
Cerebrovascular disease	99 (18.8)
Metabolic/endocrinological diseases	200 (37.2)
Diabetes mellitus	140 (26.6)
Dyslipidaemia	92 (17.5)
Respiratory diseases	81 (15.1)
Cancer	176 (33.4)
Dependence in activities of daily living (N, %)	292 (54.4)

Records indicated that 59.4% (N= 319) of septic patients had cardiovascular conditions, mainly arterial hypertension (43.5%; N=229). This was followed by neurological (39.9%, N=214) and metabolic/endocrinological diseases (37.2%, N=200), especially diabetes mellitus (26.6%, N=140). Furthermore, 54.4% (N=292) of patients were reported to experience some degree of difficulty in performing daily activities.

Table 3 summarizes the results of the pre-hospital assessment of patients. Regarding symptoms suggesting the source of infection, respiratory symptoms were the most common (50.7%, N=272), followed by impaired consciousness (30.9%, N=166) and urinary symptoms (6.7%, N=36).

**Table 3:** Summary of septic patients’ assessment Descriptive data has been represented as N, %. Serum lactate is expressed as mmol/L. Arterial partial pressure of gases is presented in mmHg.

Variable	
Suspected symptom/ sign of infection (N, %)	
Cough/ dyspnoea	272 (50.7)
Altered mental status	166 (30.9)
Dysuria/ polyakiuria	36 (6.7)
Abdominal pain	21 (3.9)
Clinical criteria determined by the physician	19 (3.5)
Cutaneous inflammatory signs	11 (2.0)
Headache	6 (1.1)
Low back pain	4 (0.7)
Jaundice	2 (0.4)
Peripheral Oxygen Saturation (N, %)	
SpO₂<90% 1^st ^assessment	268 (49.9)
SpO₂≥90% last assessment	413 (76.9)
Blood Pressure, total evaluations (N, %)	1410
<91 mmHg	889 (63.0)
91-100 mmHg	83 (13.0)
101-110 mmHg	109 (7.7)
>111 mmHg	229 (16.2)
BP, Average	99.9
Average for hypotensive patients with BP<90 mmHg	66.7
NEWS score, total evaluations (N, %)	1091
First evaluation	520 (96.8)
>1 evaluation	338 (62.9)
NEWS score ≥ 7	973 (89.2)
NEWS score, average	11.74
Blood gas analysis (N, %)	9 (1.68)
Serum lactate > 2	8 (88.9)
Serum lactate, average	3.8
Arterial partial pressure of O₂, average	58.4

An assessment of patients’ vital signs and consciousness generated at least one NEWS score in 96.8% (N=520) of cases, with an average of 11.74 points. Initial evaluation data showed that 49.9% (N=268) of patients experienced low peripheral oxygen saturation (SpO₂). Additionally, the average systolic blood pressure (sBP) recorded during the first evaluation was 99.9 mmHg, with 71.0% (N=371) of patients presenting low sBP (<90 mmHg). Blood gas analysis was conducted in only 9 (1.49%) patients, revealing an average arterial partial pressure of O₂ of 58.4 mmHg and a serum lactate level of 3.8 mmol/L.

Table 4 provides an overview of the therapeutic interventions performed by EMS teams on septic patients. Records indicated that 81.8% (N=292) of the patients assessed by the ALS and ILS teams (55.0% of all alerts) underwent vascular catheterization, whereas 11.2% (N=40) had mechanical ventilation, with 2.0% (N=7) being invasive.

**Table 4:** Description of therapeutic approaches and hospital delivery of septic patients Descriptive data have been represented as N, %. 1 – This percentage refers to the patients with low SpO2, representing 46,2% of all patients; 2 – This percentage corresponds to patients receiving specific drugs, within the sample of those treated by ALS/ILS teams (N=357). 3 - This percentage refers to the patients who received fluid therapy and had low sBP; 4 – This percentage refers to the proportion of fluids (N=265) administered within 15 minutes of the first patient assessment. NSAIDs: nonsteroidal anti-inflammatory drugs; sBP: systolic blood pressure

Variable	
Procedures (N, %)	
Vascular access	292 (54.4)
Temperature control	269 (50.1)
Non-invasive ventilation	33 (6.1)
Invasive ventilation	7 (1.3)
Therapeutic approach (N, %)	
Oxygen (when SpO_2_<90%)	248 (92.5)^1^
Fluids overall	265 (74.2)^2^
Fluids (when sBP<90 mmHg)	262 (68.8)^3^
Fluids given <15 minutes mark	76 (28.7)^4^
Fluid choice (N, %)	
Normal saline	237 (89.4)
Glucose-containing fluids	15 (5.7)
Balanced fluids	12 (4.5)
Colloids	2 (0.8)
Anti-pyretic drugs, overall (N, %)	187 (52.4)^2^
Paracetamol	177 (49.6)
Others (NSAIDs, metamizole/ dipyrone)	10 (2.8)
Vasopressors, overall (N, %)	14 (3.9)^2^
Adrenaline	7 (50.0)
Noradrenaline	4 (28.6)
Ephedrine	2 (14.3)
Phenylephrine	1 (7.1)
Hospital delivery, type (N, %)	
Polyvalent emergency department	237 (44.1)
Medical-surgical emergency department	244 (45.4)
Basic emergency department	54 (10.1)
Refused hospital transportation	2 (0.4)
Hospital delivery, place (N, %)	
Emergency department	448 (83.4)
Intensive care unit (ICU)	9 (1.7)
Non specified	82 (15.3)

Oxygen therapy was provided in 92.5% (N=248) of hypoxic patients, with 76.9% (N=413) of all patients recording SpO₂ levels above 89% in the final vital signs assessment. Antipyretics were administered in 52.4% (N=187) of patients, mostly paracetamol (94.7%, N=177 of all antipyretics).

Among all sepsis alerts, 49.3% (N=265) of patients received intravenous fluids, accounting for 74.2% of those approached by the ALS and ILS teams. As such, 33.9% (N=121) of the patients managed by these teams had a systolic blood pressure (sBP) ≥ 90 mmHg in the last recorded vital signs. The fluids used were mainly normal saline solution in 89.4% (N=237) of patients. The registration of fluid administration within the first 15 minutes on the scene was made in 28.7% (N=76) of the patients. Vasopressors were administered to 2.5% (N=14) of all patients, representing 3.9% of those managed by ALS/ILS teams. Noradrenaline was used in 28.6% (N=4) of these cases.

Patient handover primarily took place in polyvalent and medical-surgical hospitals, representing 89.6% (N=481) of cases. This distribution was influenced by the availability of local healthcare services and their proximity. The majority of patients (84.0%, N=448) were taken directly to the emergency department, while 1.7% (N=9) were admitted straight to the ICU.

Lastly, analyzing the temporal progression of each sepsis case, the median time from symptom onset to the emergency call was 90 minutes. Considering the emergency call to be time zero, the median EMS team arrival time was 26 minutes, followed by a median on-scene duration of 28 minutes. The median time to hospital arrival was 85 minutes, with only 7.6% (N=41) of cases reaching the hospital within the 60-minute mark.

## Discussion

Given the critical nature of time in sepsis treatment, it is essential to understand the pre-hospital environment. Enhancing care begins with understanding current EMS practices for sepsis patients, which is the primary goal of this study.

Our analysis shows that the gender distribution aligns with other international studies [5,7]; however, our cohort is generally older than those reported in the literature [5,7,10,11]. Our population’s medical history reveals that, as in other studies, cardiovascular diseases are the most common health issues [2]. Another significant finding is the fact that 54.4% (N=292) of the population had some degree of limitation in performing daily activities. This aligns with the observation that 28.7% (N=154) of the population was either institutionalized in an elderly facility or hospitalized, highlighting the complexity of these patients, the pathogens involved, and the resources required to treat them.

In Portugal's pre-hospital care, the NEWS score of sepsis patients recorded in iTeams™ reflects recent findings indicating that a high NEWS score leads to more admissions to the ICU and correlates with increased mortality rates [3,4] By automating the NEWS score, based on vital signs and consciousness level, the iTeams™ system enhances its reliability and efficacy as a risk assessment tool, ensuring adherence to best practices. The score was available in 96.8% (N=520) of cases, with an average score of 11.74 points. As expected, respiratory symptoms were the most prevalent, as reported in most studies [2,7,12].

Considering our telephonic triage algorithm (TETRICOSY^TM^), we found that 76.9% (N=413) of sepsis alerts were rated P1, leading to ALS or ILS dispatch in 15.9% (N=85) and 50.7% (N=272) of cases, respectively. In 10.3% (N=56) of the cases, despite the team assigned based on the triage algorithm being ALS/ILS, a BLS team was dispatched. This occurs in one of two scenarios: either the local ALS/ILS teams are unavailable or the transport by a BLS team to the nearest hospital was faster than waiting for an ALS/ILS team on-site. These findings might suggest a selection bias, as either the identified population was critically ill or the telephonic triage algorithm classifies them as such [7].

While point-of-care lactate testing does not predict ICU admissions, it is known that its measurement significantly reduces the time required for IV fluids and antibiotic administration, both inside the hospital and in the pre-hospital setting [2,13,14]. The limited number of ALS teams with access to the necessary equipment for serum lactate testing contributes to the low frequency of lactate and arterial blood gas assessments, which hinders proper analysis. However, in comparison to other studies, a larger number of patients exhibited lower sBP [6,7]. This aligns with the established understanding that EMS septic patients tend to be more severely affected than those in hospitals [14].

Considering the procedures and therapeutic approaches, 81.8% (N=292) of ILS/ALS teams performed vascular catheterization of any kind. Nevertheless, this number seems low given the percentage of hypotensive patients (70.9%, N=381) and the recommended fluid challenge regardless of sBP value. However, these results may be biased due to incomplete or inaccurate documentation.

Regarding supplemental oxygen, its effect is evident in 76.9% (N=413) of patients with SpO₂ greater than 89% in the final evaluation. However, there is missing data from a second evaluation in 7.5% (N=20) of hypoxic patients. Since the registration typically occurs after the patient has been stabilized and the procedures completed, we believe some details may be overlooked when filling out the appropriate checkboxes within the clinical record. This becomes apparent when examining specific charts showing IV fluid administration but lacking corresponding IV catheterization records.

Although the literature supports the use of balanced fluids in sepsis [15], it is well-recognized that the adverse effects of saline regarding hyperchloremic acidosis occur with larger volumes of fluids such as those found in the ICU [16]. Fluids were administered in 74.2% (N=265) of ALS/ILS-handled patients, roughly half the population. Normal saline was the most commonly used fluid in 89.4% (N=237) of patients; balanced fluids were only used in 4.5% (N=12), and a small percentage of colloids was utilized (0.9%, N=2). Prehospital care should be a swift component of the sepsis process, so choosing a normal saline solution may not be totally wrong. This logic also applies to vasopressor use; they should be administered when the patient has received adequate fluid resuscitation, which takes some time, so we are not surprised by the small percentage of vasopressors administered in our EMS context.

It is also important to consider the time required for IV fluid administration, with 28.8% (N= 76) of patients receiving fluids in less than 15 minutes. Based on previous clinical documentation, we believe there may be data inaccuracies due to typical delays in registration and the assumption by the computer system that the registration time matches the procedure time. According to the state-of-the-art and ongoing training given to EMS teams, once sepsis is identified and the IV/IO catheter placed, fluid resuscitation is initiated immediately. Consequently, we anticipated that this value would be significantly higher than what was observed.

While many pre-hospital and hospital studies highlight the benefits of early antibiotic administration [1,2,6,7,13,14], it is important to emphasize that our pre-hospital teams do not have access to these medications. As such, we acknowledge that it is a major limitation of our team's approach to sepsis management.

Finally, the handover of patients with suspected sepsis is managed according to national health protocols, with admission typically occurring in the emergency department or directly to the intensive care unit. Territorial disparities, particularly in the interior regions of Portugal, pose logistical challenges that contribute to delays. The distance to hospitals, along with inadequate road infrastructure for high-speed travel, helps explain the median hospital arrival time of 85 minutes. Nevertheless, recent initiatives, such as campaigns aimed at healthcare professionals, have been implemented to reduce the time spent on approaching the patient before transportation, ensuring that antibiotics are administered within the first 60 minutes.

The limitations of our study stem from its retrospective design, relying on clinical records that may contain missing or inaccurate information. Therefore, we can not generalize our findings. On the other hand, as our study operates within a national context, it is nearly impossible to know what happened to the patients after they were handed over to the hospital. In Portugal, although the EMS software application is linked to the public hospital network, clinical records are kept on different platforms, making it difficult to access intra-hospital patient information.

## Conclusions

Pre-hospital sepsis management is challenging, both in terms of diagnosis and ensuring timely interventions. However, simple strategies, like standardized training and frequent updates, could significantly enhance the care provided to these patients. We believe that our pre-alert system is adequate but lacks evidence of its effectiveness. This can be addressed through two main strategies: First, establishing partnerships to share information between INEM and the hospital-based sepsis teams that receive these patients, enabling EMS to audit its performance, through data as ward or ICU admission and mortality rates. Second, it is crucial to expand the use of iTeams™ to all EMS teams to ensure that all sepsis alerts can be equally analyzed. A major challenge for EMS is the difficulty of providing antibiotics in the pre-hospital setting. Even if EMS teams could successfully identify all septic patients and provide the appropriate fluid resuscitation and organ support, our ability to meet the critical time window for antibiotic administration still depends on how quickly we can transport patients to a hospital.

This study opens up new avenues for further research into the approach to sepsis in the pre-hospital setting, particularly in determining the best criteria for the alert system, and the benefits of lactate determination and antibiotic administration before arrival at the hospital.
